# Characterization of ALBA Family Expression and Localization in *Arabidopsis thaliana* Generative Organs

**DOI:** 10.3390/ijms22041652

**Published:** 2021-02-06

**Authors:** Alena Náprstková, Kateřina Malínská, Lenka Záveská Drábková, Elodie Billey, Dagmar Náprstková, Eva Sýkorová, Cécile Bousquet-Antonelli, David Honys

**Affiliations:** 1 Laboratory of Pollen Biology, Institute of Experimental Botany of the Czech Academy of Sciences, Rozvojová 263, 165 02 Prague 6, Czech Republic; naprstkova@ueb.cas.cz (A.N.); l.zaveska.drabkova@ueb.cas.cz (L.Z.D.); dagmar.naprstkova@seznam.cz (D.N.); 2 Imaging Facility, Institute of Experimental Botany of the Czech Academy of Sciences, Rozvojová 263, 165 02 Prague 6, Czech Republic; malinska@ueb.cas.cz; 3 CNRS LGDP-UMR5096, 58 Av. Paul Alduy, 66860 Perpignan, France; Elodie.Billey@cea.fr (E.B.); cecile.antonelli@univ-perp.fr (C.B.-A.); 4 LGDP-UMR5096, Université de Perpignan via Domitia, 58 Av. Paul Alduy, 66860 Perpignan, France; 5 Institute of Biophysics of the Czech Academy of Sciences, Královopolská, 612 00 Brno, Czech Republic; evin@ibp.cz

**Keywords:** *Arabidopsis thaliana*, ALBA, flowering, pollen development, heat stress, expression analysis, protein localization, confocal microscopy, PABP3

## Abstract

ALBA DNA/RNA-binding proteins form an ancient family, which in eukaryotes diversified into two Rpp25-like and Rpp20-like subfamilies. In most studied model organisms, their function remains unclear, but they are usually associated with RNA metabolism, mRNA translatability and stress response. In plants, the enriched number of ALBA family members remains poorly understood. Here, we studied *ALBA* dynamics during reproductive development in *Arabidopsis* at the levels of gene expression and protein localization, both under standard conditions and following heat stress. In generative tissues, ALBA proteins showed the strongest signal in mature pollen where they localized predominantly in cytoplasmic foci, particularly in regions surrounding the vegetative nucleus and sperm cells. Finally, we demonstrated the involvement of two Rpp25-like subfamily members ALBA4 and ALBA6 in RNA metabolism in mature pollen supported by their co-localization with poly(A)-binding protein 3 (PABP3). Collectively, we demonstrated the engagement of ALBA proteins in male reproductive development and the heat stress response, highlighting the involvement of ALBA4 and ALBA6 in RNA metabolism, storage and/or translational control in pollen upon heat stress. Such dynamic re-localization of ALBA proteins in a controlled, developmentally and environmentally regulated manner, likely reflects not only their redundancy but also their possible functional diversification in plants.

## 1. Introduction

ALBA-family (Acetylation lowers binding affinity) proteins belong to an ancient group of proteins found in all domains of life [1,2]. All members of the family are characterized by a highly conserved nucleic acid-binding ALBA domain, possessing a IF3-C fold with RNA-binding properties [1]. ALBAs are small basic proteins well known to form dimers [1,2]. In the eukaryotic lineage, these proteins diversified into two subfamilies, the Rpp25-like subfamily, which includes shorter genes, and the Rpp20-like subfamily, which comprises longer genes possessing RNA-binding RGG repeats [1]. In humans, each subfamily consists of a sole member, RPP20 and RPP25, respectively. They dimerize in the nucleolus as a component of RNase P, a multi-subunit enzyme involved in tRNA maturation [1,3,4,5]. In protozoan parasites, ALBA proteins are encoded by a single gene within each subfamily, or, by small gene families encoding proteins predominantly localized in the cytoplasm under standard conditions. They are associated with RNA metabolism and translation [6,7,8].

In plants, ALBA homologues have been investigated in several species. Seed plant genomes underwent genome duplications and rearrangements which led to the gene duplications observed in the ALBA family [9,10]. In rice (*Oryza sativa*), nine *ALBA* genes were identified, showing tissue-specific expression profiles, with expression also detected in generative organs [10]. Both ALBA subfamilies are equally represented, comprising four members, *OsALBA1, OsALBA2, OsALBA6,* and *OsALBA9* in the Rpp25-like subfamily, and *OsALBA3, OsALBA4, OsALBA5,* and *OsALBA8* in the Rpp20-like subfamily [10,11]. *OsALBA7* has a different domain composition, not exactly falling within these two sub-families [10,11]. In *Arabidopsis thaliana*, six homologues are equally shared by the Rpp20-like (AtALBA1, AtALBA2, and AtALBA3/DAN1) and Rpp25-like (AtALBA4, AtALBA5, and AtALBA6) subfamilies [10,12]. Each subfamily, Rpp20-like and Rpp25-like, contains two close paralogs (AtALBA1 and AtALBA2, AtALBA4 and AtALBA5) and one more distantly related member AtALBA3 and AtALBA6, respectively. Their expression profiles were studied in rosettes, roots and flower buds, where all *ALBA* genes were preferentially active in young developing tissues including root tips, cotyledons, and leaf primordia. *AtALBA1* and *AtALBA4* showed the highest expression in all observed tissues within Rpp20-like and Rpp25-like subfamilies while *ALBA3/DAN1* was specifically expressed in flowers [12,13]. Available localization data have shown that AtALBA1, AtALBA2, AtALBA4, and AtALBA5 localize to the cytoplasm in root tips [12], while OsALBA1-GFP, transiently expressed in onion epidermal cells, localize to the nucleus and the cytoplasm [11].

Various stress conditions have been shown to cause changes in ALBA behavior. In *Plasmodium falciparum*, ALBA homologues associate with factors that regulate translation repression [7]. In *Leishmania infantum*, temperature stress caused ALBA protein (LiAlba1/Rpp20 and LiAlba3/Rpp25) re-localization from the cytoplasm into the nucleolus and flagellum, together with PABPs [6]. Similarly, *Trypanosoma brucei* ALBAs were detected in association with mRNAs, exclusively in cytoplasmic stress granules following nutrient stress [7,8]. In plants, ALBA proteins have also been connected to various stress responses [10,14,15]. In rice seedlings, significant changes in the relative expression of *ALBA* genes were demonstrated following various abiotic stress and phytohormone treatments [10]. Most genes (across subfamilies) showed differential expression following heat stress application (42 °C); *OsALBA1, OsALBA2, OsALBA6, OsALBA7,* and *OsALBA8* were upregulated, while *OsALBA4* was downregulated [10]. Similarly, differential expression upon abiotic stress application (water and salt stress) was observed for *ALBA* genes in *Gossypium hirsutum* (cotton) leaves, roots, and stems. In leaves and roots, two genes *GhALBA_4* and *GhALBA_5* were significantly upregulated [14]. Transgenic plants with reduced expression of these genes were shown to be more sensitive to drought and salt stress [14]. Moreover, *AtALBA1*, *AtALBA2*, *AtALBA4*, *AtALBA5,* and *AtALBA6* enrichment was detected in the mRNA binding proteome of *Arabidopsis* four-day-old etiolated seedlings [15].

Increased temperatures often has a harmful effect on plant reproduction and fertility. Reproductive development is particularly sensitive to heat stress treatment [16]. The male gametophyte, pollen grain, plays a critical role in the plant reproduction process by the formation and delivering the male sperm cells to the female gametophyte to achieve double fertilization [17]. High temperatures can cause asynchrony of male and female gametophyte development, ovule number reduction and abortion, aborted pollen development, lower pollen germination ability, and, as a consequence, reduced plant reproduction fitness [18,19]. Considering the stress sensitivity of reproductive organs and the modulation of *ALBA* expression by various abiotic stresses, we studied the expression dynamics of all *ALBA* genes in *Arabidopsis* generative tissues and investigated the response of a subset of them to heat stress.

## 2. Results

### 2.1. Expression Analysis of ALBA Genes in Arabidopsis Inflorescences

**Figure 1:**
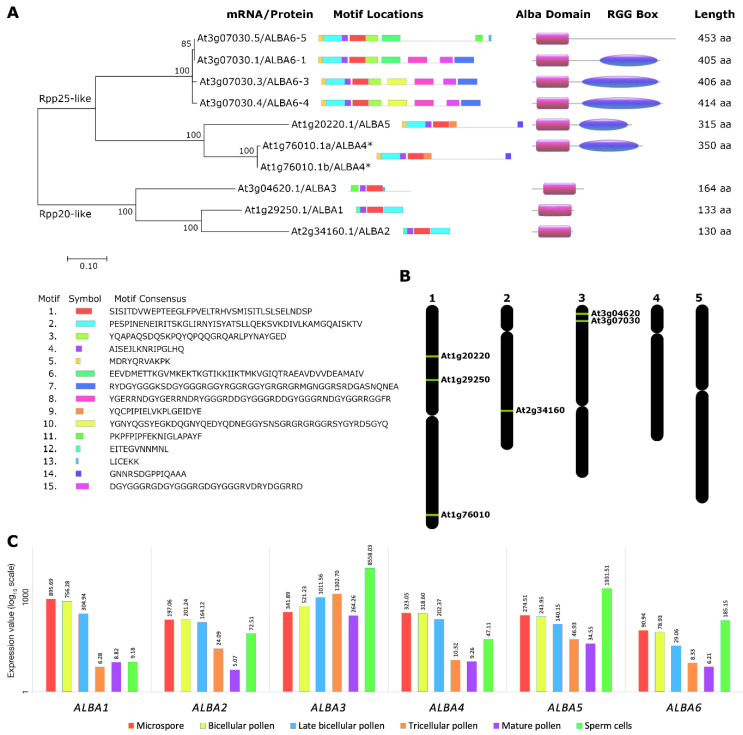
Schematic overview of ALBA proteins during *Arabidopsis* male gametophyte development. (**A**) Maximum likelihood (ML) unrooted tree showing the two ALBA subfamilies and their mRNAs. The bootstrap support values are shown above the branches (ML log likelihood—4274.4785). The tree is drawn to scale, with branch lengths measured in the number of substitutions per site. Obtained sequences of ALBA-family cDNA from inflorescences were used for conserved motifs identification and aligned to known structures from UniProt. Individual domains in ALBA proteins were identified by CATCH online tool. RGG box represents summary of RGG boxes found in the primary sequences (Table S1). * At1g76010.1 encodes two mRNA variants with 5´UTR and 3´UTR sequence variability. (**B**) Representation of *ALBA* genes layout on *Arabidopsis* chromosomes. (**C**) A comparison of *ALBA* transcription levels in developing pollen and sperm cells based on RNA-seq data at logarithmic scale (log_10_).

The *Arabidopsis* ALBA family is well conserved and comprises six homologs. The *AtALBA* genes form two subfamilies (Figure 1A). The Rpp20-like subfamily comprises shorter genes (*ALBA1* (At1g29250), *ALBA2* (At2g34160) and *ALBA3* (At3g04620)) possessing only an ALBA domain, whereas the Rpp25-like subfamily contains longer genes (*ALBA4* (At1g76010), *ALBA5* (At1g20220) and *ALBA6* (At3g07030)) carrying an ALBA domain and a RGG rich carboxyterminal extension (Figure 1A). The *AtALBA* genes are unequally distributed on three chromosomes, with Rpp20-like subfamily members on chromosomes 1, 2, and 3 and Rpp25-like subfamily members on chromosomes 1 and 3 (Figure 1B) [12]. *ALBA1*, *ALBA2*, *ALBA3*, and *ALBA5* are represented by a single mRNA, whereas *ALBA4* produces two similar mRNAs (At1g76010.1a and At1g76010.1b) with different untranslated regions (5’UTR and 3´UTR). *ALBA6* gene on the other hand produces four splice variants *ALBA6-1* (At3g07030.1), *ALBA6-3* (At3g07030.3), *ALBA6-4* (At3g07030.4), and *ALBA6-5* (At3g07030.5). These sequences were analyzed and their relations compared using a maximum likelihood unrooted tree construction (Figure 1A). A detailed sequence analysis revealed conserved motifs that are common for all members (motif 1 and 4), conserved within a subfamily (motif 5), common for the closest paralogs in each subfamily (motif 9, 14 and 12), and common for the most divergent homologs in each subfamily (motif 5 and 11). Interestingly, the position of motif 2 is stable in the Rpp25-like subfamily, whereas it varies in the Rpp20-like subfamily members ALBA1 and ALBA2. Moreover, motif 2 is absent in ALBA3.

Following the analyses of the *ALBA* gene family, we next sought to characterize the respective genes in the generative organs of *Arabidopsis*, particularly the developing male gametophyte. We analyzed the abundance of *ALBA* mRNAs during crucial phases of pollen development (e.g., microspores, bicellular pollen, late bicellular pollen, tricellular pollen, mature pollen) and in sperm cells (Figure 1C). With the exception of *ALBA3*, the expression of all other *ALBA* genes declined from microspores to mature pollen. This decline was either gradual (*ALBA2, ALBA5,* and *ALBA6*) or sharp between bicellular and tricellular pollen (*ALBA1* and *ALBA4*). In contrast to these genes, *ALBA3/DAN1* expression, activated by the DUO1 transcription factor [13], shows an opposite trend, with transcription increasing towards tricellular pollen followed by its decrease in mature pollen. Moreover, a considerable enhancement in the expression of *ALBA2-ALBA6* was detected in sperm cells at the mature pollen stage. This enhancement was particularly strong for *ALBA3/DAN1*.

To investigate *ALBA* expression more closely, *ALBA* promoter regions were fused to the β-glucuronidase (GUS) reporter gene and their activity documented in transformed (*ALBA1-6*) and untransformed Col-0 plants. T1 generation plants were screened for GUS expression with at least two independent lines with a representative and stable GUS expression pattern selected per construct. During reproductive development, various patterns of expression were found within the *ALBA* family. We observed similarities between both subfamilies. In each subfamily, there was one dominantly expressed gene (ALBA1—Rpp20-like and ALBA4—Rpp25-like) with the remaining two genes showing weaker expression.

**Figure 2:**
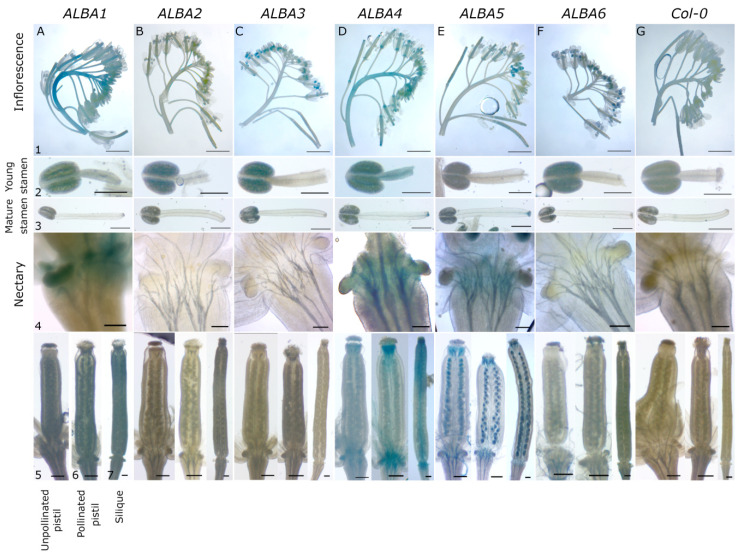
Activity of *ALBA* promoters in *Arabidopsis* inflorescences and flowers. *Arabidopsis* plants harboring *ALBA* promoters (*ALBA1* (**A**), *ALBA2* (**B**), *ALBA3* (**C**), *ALBA4* (**D**), *ALBA5* (**E**), *ALBA6* (**F**)) driving the β-glucuronidase (*GUS)* marker gene, and wild type Col-0 plants (**G**) were used for promoter activity assays. Whole inflorescences were collected and stained for GUS activity. Young anthers (2) and pistils (5) were collected from flower stage 12, mature stamens (3) and pistils (6) originated in flower stage 13, and mature siliques (7) were collected from flower stage 17 [20]. Scale bars: inflorescences, 500 µm (1); stamens and nectaries, 100 µm (2, 3, 4); pistils and siliques, 50 µm (5, 6, 7).

The *ALBA1* promoter is highly active in inflorescence stems, flower buds, and developing and maturing flowers (Figure 2A1). Moreover, a GUS signal was detected in anther tapetum but not in the filaments of young stamens (Figure 2A2). In open flowers, the signal from mature anthers disappeared (Figure 2A3), however, significant *ALBA1* promoter activity was found at the receptaculum and nectaries (Figure 2A4). Finally, weak promoter activity was observed in young pistils before maturation. It became stronger after pollination at the base, in the style and in the carpels, with the GUS signal finally increased at distal parts of siliques (Figure 2A5–A7).

*ALBA2* and *ALBA3* promoters are active exclusively in pollen, with *ALBA3* being stronger. Both promoters were more active in young anthers, with the signal diminished at pollen maturity. Neither *ALBA2* nor *ALBA3* promoters were active in other flower tissues (Figure 2B1–C7).

*ALBA4* promoter activity was similar to that of *ALBA1*. Significant GUS signal was identified in inflorescence stems, buds and receptaculum following pollination (Figure 2D1). GUS signal was present in developing stamen in tapetum and filament (Figure 2D2) but at the stamen maturity it remained only at filament base (Figure 2D3). In flower receptaculum, the GUS signal was detected in nectaries (Figure 2D4). Changes in expression were observed in the course of pistil and silique development, where a weak *ALBA4* expression signal appeared at distal parts of young pistils (including nectaries) and disappeared towards the middle part. Moreover, *ALBA4* activity becomes stronger during pistil maturation and its conversion into a silique, where it unequally expands from distal areas towards the center with the only signal connection in septum (Figure 2D5–D7). *ALBA5* and *ALBA6* promoter activity were weaker than *ALBA4′*s. *ALBA5* expression was more targeted to young buds, with a weak GUS signal in developing flowers becoming more intense in siliques (Figure 2E1). There was no visible signal in young stamens (Figure 2E2), whereas we detected a weak GUS signal at the base of mature filaments (Figure 2E3). Weak *ALBA5* promoter activity was observed at receptaculum but not in nectary (Figure 2E4). Significant expression was detected in distal parts of developing pistils and siliques, and, interestingly in unfertilized and fertilized ovules, and in developing seeds (Figure 2E5–E7). Finally, weak pollen-specific *ALBA6* promoter activity was detected exclusively in developing, but not mature, anthers (Figure 2F1–F3).

**Figure 3:**
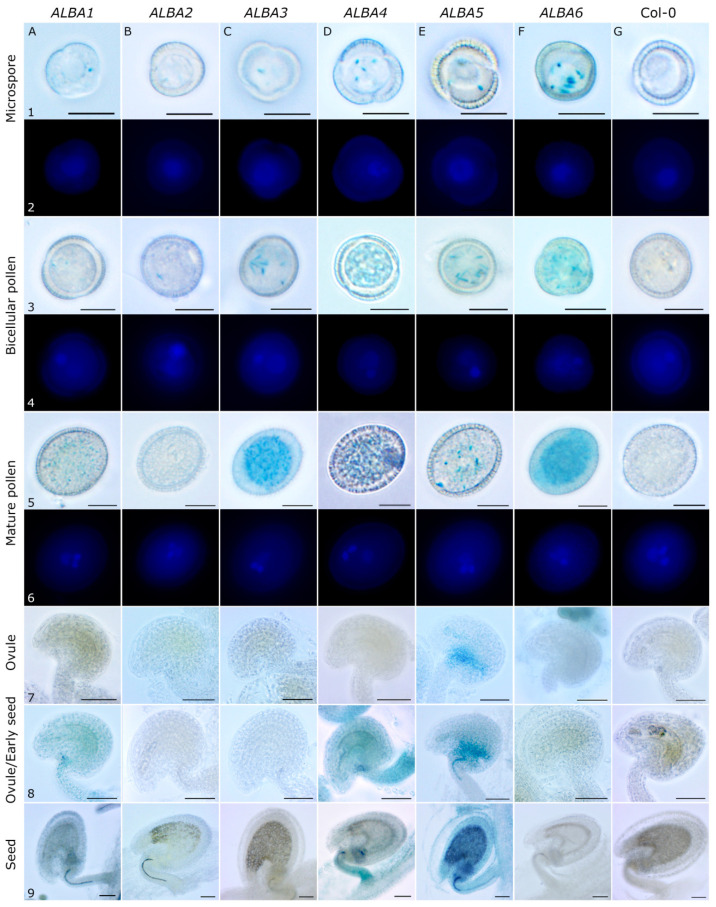
Activity of *ALBA* promoters in *Arabidopsis* male gametophyte and ovules/developing seeds. The Arabidopsis plants harboring *ALBA* promoters (*ALBA1* (**A**), *ALBA2* (**B**), *ALBA3* (**C**), *ALBA4* (**D**), *ALBA5* (**E**), *ALBA6* (**F**)) driving GUS marker gene, and wild type Col-0 plants (**G**) were used for promoter activity assays. Whole inflorescences were collected and stained for GUS activity. From them, pollen at three developmental stages, microspores (1, 2), bicellular pollen (3, 4), and mature pollen (5, 6) were isolated as well as ovules from unpollinated pistils (7), ovules from pollinated pistils (8), and developing seeds (9). Scale bars: microspores, bicellular and mature pollen, 10 µm (1, 3, 5); ovules and seeds, 50 µm (7, 8, and 9).

The strongest activity of the studied genes was observed in the generative organs, stamens and carpels. Therefore, we decided to analyze *ALBA* promoter activities in male and female gametophytes (Figure 3). Individual gene expression patterns were investigated throughout pollen development at distinct stages (microspores, bicellular pollen, and mature pollen) (Figure 3A1–F6). In female reproductive tissues, ALBA promoter activities were examined throughout ovule and seed development in ovules from unpollinated (Figure 3A7–F7) and pollinated pistils (Figure 3A8–F8), and in developing seeds (Figure 3A9–F9).

*ALBA1* and *ALBA3-ALBA6* promoters (Figure 3A1–F1) were active already in microspores, as verified by DAPI staining (Figure 3A2–G2). their expression pattern did not change after pollen mitosis I (PMI) since the respective GUS signal persisted in the bicellular pollen (Figure 3A3–G4). On the contrary, the *ALBA2* promoter-driven GUS signal only starts appearing at the bicellular pollen (Figure 3B3,B4). Closer to the maturity, *ALBA* expression patterns become more distinct. There, pollen-specific *ALBA3* and *ALBA6* are the most active promoters, followed by weaker *ALBA1* and *ALBA4* and the weakest *ALBA5* (Figure 3A5–G6). *ALBA2* expression was not detected.

*ALBA* promoter activity in ovules almost mirrored their activity in pollen (Figure 3A7–F9). *ALBA3* and *ALBA6*, with the strongest promoter activity in pollen, showed no activity in ovules. *ALBA1* and *ALBA4* promoters, moderately active in pollen, were moderately strong in ovules. Finally, the weakest mature pollen-active promoter, *ALBA5*, was the strongest, and the earliest, during ovule development. In fact, *ALBA5* was the only *ALBA* promoter active in ovules within unpollinated pistils. We detected its activity preferably in the chalazal region near the funiculus, and a very weak signal at the micropylar pole of the female gametophyte (Figure 3E7). This pattern persisted and spread in ovules from pollinated pistils (Figure 3E8). Interestingly, *ALBA1* and *ALBA4* promoter activity in pollinated ovules and developing seeds localized mainly in funiculus around vascular tissues and weakly in female gametophytes/seeds at their chalazal end (Figure 3A8,A9,D8,D9). Only the *ALBA5* promoter was active in the whole developing seed (Figure 3E9).

### 2.2. Subcellular Localization of ALBA Proteins during Pollen Development

To investigate the subcellular localization of studied proteins during pollen development, we generated transgenic plants expressing ALBA-GFP fusion proteins. Genomic fragments were used for *ALBA1-ALBA5*, while *ALBA6* was amplified from pollen cDNA. Their expression was driven by the respective native promoters. All ALBA proteins possess a globular N-terminal ALBA domain, and a heterogenic C-terminal part that was selected for the GFP fusion.

**Figure 4:**
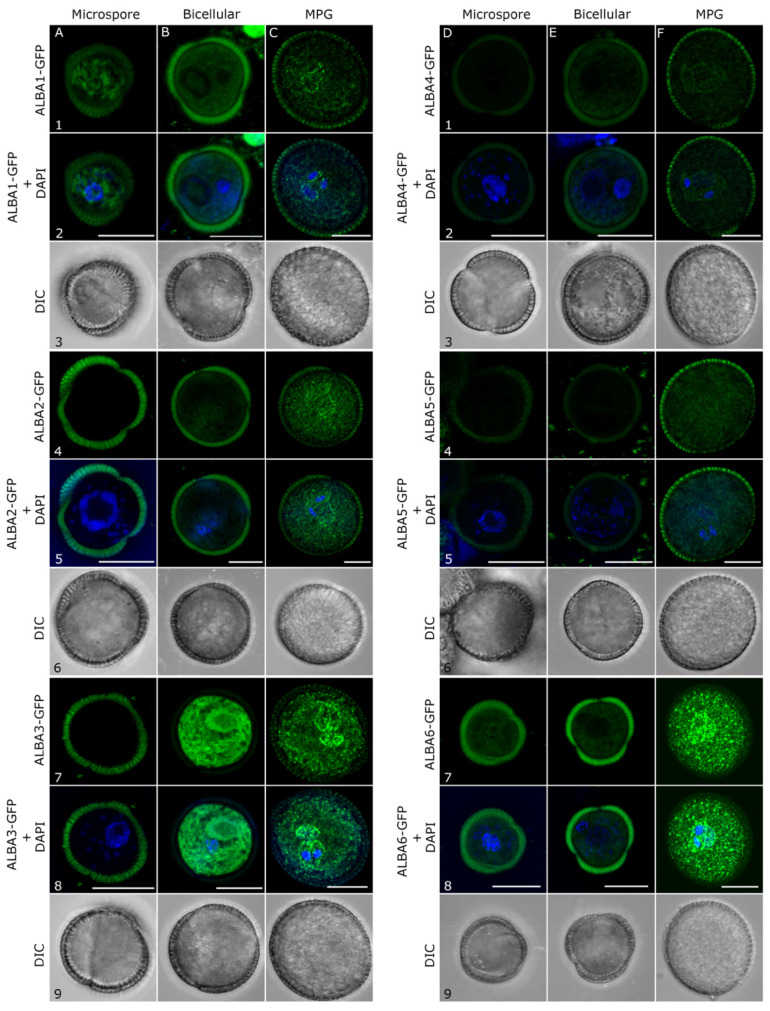
Subcellular localization of ALBA-GFP fusion proteins in transgenic plants during pollen maturation. Genomic DNA fragments of Rpp20-like subfamily (ALBA1, ALBA2, and ALBA3) and Rpp25-like subfamily (ALBA4 andALBA5) and cDNA encoding the pollen-expressed ALBA6-4 isoform were fused with GFP (C-terminal fusions) and expressed under their native promoters. Isolated stages of pollen development were stained with DAPI for nuclei visualization. Scale bars = 10 µm.

In microspores, only *ALBA1* and *ALBA6* GFP fusions were detected, with both localizing to the cytoplasm. ALBA6-GFP localized homogenously in the cytoplasm (Figure 4D7,D8), while ALBA1-GFP was found in pronounced elongated foci (Figure 4A1,A2). These two proteins showed similar, generally non-specific, cytoplasmic localization in bicellular pollen (Figure 4B1,B2,E7,E8). The fluorescence signal of ALBA2-GFP (Figure 4B4,B5) and ALBA4-GFP (Figure 4E1,E2) was localized in a small number of round and elongated foci, whereas ALBA3-GFP presented the dominant ALBA protein expressed in the bicellular pollen. It was detected in a high number of elongated foci/fibres in the cytoplasm and found to be enriched around the vegetative nucleus (Figure 4B7,B8). Finally, all ALBA-GFP proteins were detected in mature pollen, being the only stage where we detected ALBA5-GFP. *ProALBA6::GFP-GUS* transgenic plants were used as a positive control for cytoplasmic protein localization while wild type Col-0 plants served as a negative control (Figure S1).

All ALBA proteins showed strongest expression in mature pollen, with fluorescence detected in the cytoplasm of both cell types forming the male gametophyte—the vegetative cell (VC) and sperm cells (SC) (Figure S2). In the male germ unit (MGU), a reticular-like signal distribution was enriched by a high number of distinct foci that were concentrated in the proximity of vegetative nuclei (VN) and sperm cell membranes (Figure 4). ALBA3-GFP and ALBA6-GFP were the most abundant ALBA proteins in mature pollen (Figure 4), although their expression signal at the RNA level (Figure 1) was not the highest there. It suggests their possible regulation also at post-transcriptional levels. They both represent the most divergent members of their respective subfamilies. They similarly localized predominantly in enlarged particles accumulated around the MGU, likely close or in association with their membrane structures (Figure 4C7,C8,F7,F8).

Of them, ALBA3-GFP appeared to preferentially localize in the sperm cells around sperm cell nuclei. In addition to this preferential localization in proximity to the MGU, both these proteins also accumulated in distinct foci of variable size in the VC cytoplasm.

The two remaining Rpp20-like subfamily members, ALBA1-GFP and ALBA2-GFP, were less abundant. They accumulated preferentially in round foci attached to VN and in the large, elongated particles in close proximity to SC nuclei, (Figure 4C1,C2,C4,C5) as well as in numerous cytoplasmic particles at lower abundance. This localization pattern, although more diffuse in the cytoplasmic portion, was observed also for the remaining Rpp25-like subfamily members, ALBA4-GFP and ALBA5-GFP (Figure 4F1,F2,F4,F5).

### 2.3. ALBA Genes Expression in Arabidopsis Inflorescences after Heat Stress

The role of ALBAs in the stress response has been documented in several cases. Therefore, we were interested in whether *ALBA* gene expression is affected by heat stress. To this effect, Col-0 plants were exposed to mild (37 °C for 3 h) or moderate (42 °C for 1 h) heat stress, their inflorescences collected (1 h or 24 h post-treatment to distinguish early and late heat stress response) for RNA isolation and RT-qPCR subsequently carried out. Melting curve analysis consistently demonstrated a single homogenous melting peak for all primer sets (Table S2; Primers 1–16). We selected *GAPC1* (At3g04120) and *EIF1a4* (At5g60390) as reference genes for the experiments and evaluated the data (Table S3) by the Student’s t-test and the Wilcoxon test (details are presented in Table S4).

**Figure 5:**
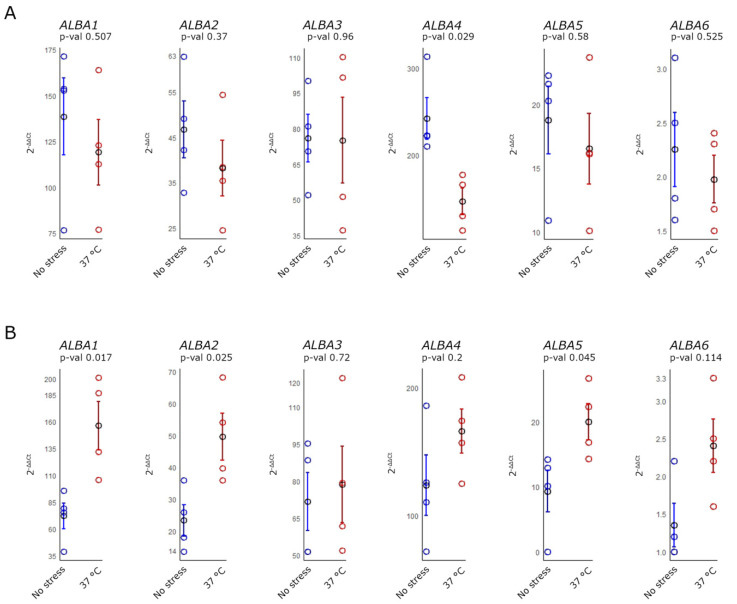
Heat stress-induced relative expression changes of *ALBA* genes in inflorescences evaluated by RT-qPCR. Flowering plants were exposed to 37 °C for 3 h; inflorescences of treated and untreated plants were collected 1 h (**A**) and 24 h after the end of the heat exposure (**B**). Relative expression values were compared with the untreated samples collected at the same time. The measurement was performed in four biological replicates and two technical replicates for each sample. All data were tested for normality and *p*-values were obtained for each experiment (see Table S4). The blue and red rings represent the individual replicates; sample averages of each independent group are represented by black rings with indicated standard error bars.

We observed significant changes in expression (*p*-value < 0.05) 1 h and 24 h after the application of 37 °C heat stress (HS) (Figure 5). All ALBA genes showed reduced expression as an early response 1 h after the treatment and increased expression later on, 24 h after HS. Average values of all *ALBA* expression data showed a declining trend in *ALBA* expression during the early response compared to the untreated controls (Figure 5A). However, most of them did not show a statistically significant difference. Only the evaluation of *ALBA4* expression (*p*-value = 0.029) revealed a statistically significant decrease after the treatment. Interestingly, *ALBA* expression increased 24 h following HS (Figure 5B). The application of 42 °C did not cause significant changes (*p*-value < 0.05) in *ALBA* expression (Figure S3). However, most of the *ALBA* genes were upregulated early after the treatment, except *ALBA6*, with decreased expression. Higher variability was observed 24 h after HS since only *ALBA3* expression decreased in comparison to the untreated control, while the expression of the other genes indicated increased expression.

### 2.4. Heat-Stress Induces Aggregation of ALBA Proteins in Pollen

**Figure 6:**
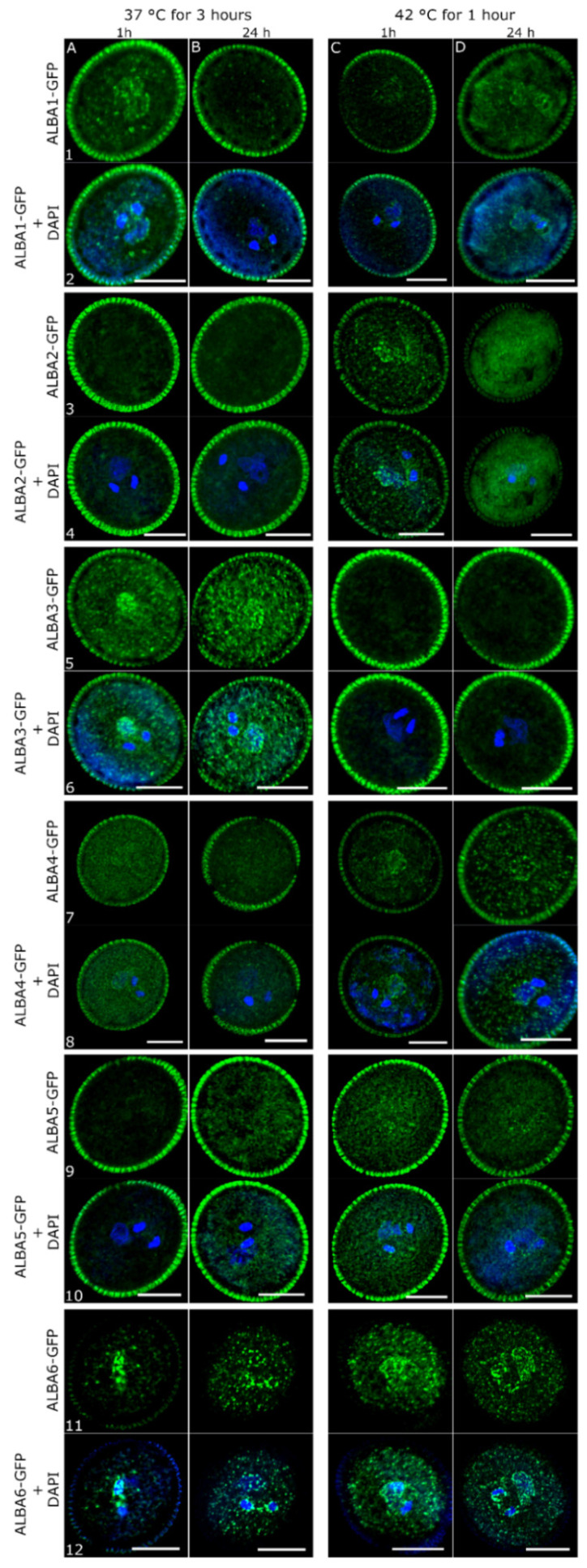
ALBA-GFP localization in pollen after heat stress. ALBA-GFP harboring plants were exposed to 37 °C for 3 h or 42 °C for 1 h. Pollen samples were collected 1 h and 24 h after the stress treatment, DAPI-stained and observed by confocal microscopy. Scale bars = 10 µm.

To investigate the heat-stress effect on ALBAs at the protein level, we applied heat stress to ALBA-GFP transgenic plants and observed mature pollen from open flowers. The same stress regimes were applied (37 °C for 3 h and 42 °C for 1 h). Mature pollen was harvested 1 h or 24 h after HS to distinguish the early and late response, respectively. As under standard conditions (Figure 4), the studied proteins generally accumulated in cytoplasmic foci of various sizes following individual treatments (Figure 6). In most samples, heat stress application led to the redistribution of ALBA-GFP fusion proteins to foci of higher fluorescence intensities, while the cytoplasmic signal decreased. Protein re-localization was more pronounced within the Rpp20-like subfamily compared to the Rpp25-like one. Cytoplasmic signal distribution in *proALBA6::GFP-GUS*-transformed pollen was not affected by heat-stress and served as a control (Figure S4).

All three Rpp20-like subfamily members showed different stress-induced localization patterns. ALBA1-GFP, less abundant in mature pollen under control conditions, increased in abundance shortly after 37 °C treatment, and accumulated around MGU in several large cytoplasmic particles, whereas the GFP signal was less abundant in pollen VC collected 24 h after the treatment (Figure 6A1–B2). Heat stress at 42 °C brings about the inverse. No ALBA1-GFP signal was detected after 1 h; however, it became more dispersed and non-specific in VC cytoplasm later on (Figure 6C1–D2). On the contrary, ALBA2-GFP almost completely disappeared after 37 °C treatment (Figure 6A3–B4), whereas it increased in abundance shortly after 42 °C treatment. Moderate stress also resulted in ALBA2-GFP accumulation around MGU and in several large and numerous small cytoplasmic particles. However, the late response of 42 °C was similar to that of ALBA1-GFP (Figure 6C3–D4).

The heat stress response of the two less abundant Rpp25-like subfamily members, ALBA4-GFP and ALBA5-GFP, was less dramatic. Neither showed enrichment around MGU under any conditions. Within the subfamily, a stronger signal accumulation in cytoplasmic foci was captured in ALBA4-GFP pollen after the 37 °C treatment (Figure 6A7–B8). A 42 °C HS treatment caused ALBA4-GFP to almost completely redistribute from the cytoplasm into cytoplasmic foci, with the late response being stronger than the early one (Figure 6C7–D8). ALBA5-GFP also accumulated in cytoplasmic foci of various sizes following both HS treatments, but with different dynamics. At 37 °C, a stronger late response was induced, whereas 42 °C led to a mildly stronger early response (Figure 6A9–D10). Therefore, ALBA4-GFP and ALBA5-GFP were the only ALBA proteins showing a stronger late response in pollen, although under different temperature conditions.

ALBA3-GFP and ALBA6-GFP, being the most abundant ALBAs in untreated mature pollen (Figure 4), both accumulated around MGU in response to 37 °C, both early and late. However, although ALBA3-GFP was specifically enriched around VN, ALBA6-GFP accumulated preferably around SC (Figure 6A5–B6,A11–B12). Interestingly, in untreated mature pollen, both these proteins showed a similar localization pattern surrounding both VN and SC and presence in foci distributed evenly in the VC cytoplasm. Both proteins tended to re-localize from close proximity to the MGU towards cytoplasmic foci, during the early and late stress response. The response of ALBA3-GFP and ALBA6-GFP to 42 °C treatment was different. ALBA3-GFP completely disappeared (Figure 6C5–D6), while ALBA6-GFP showed similar localization as observed in untreated mature pollen with an enriched signal around MGU and dispersed signal in the VC cytoplasmic foci (Figure 6C11–D12).

**Figure 7:**
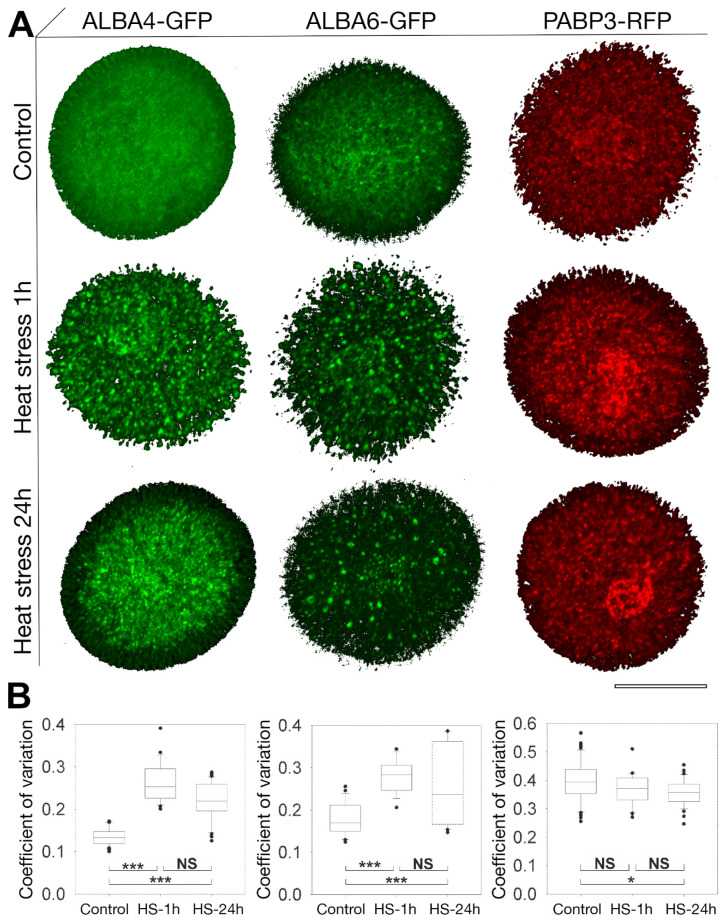
Quantification of ALBA-GFP clustering upon heat stress. (**A**) 3D-view of confocal z-stacks of ALBA4-GFP (left column), ALBA6-GFP (middle column) and PABP3-RFP (right column) expressed under native promoters at standard conditions (upper row) and 1 h (middle row) and 24 h (lower row) after the heat shock treatment (37 °C for 3 h). Scale bar 10 µm. (**B**) Coefficient of variation (CV) reflecting fluctuations in fluorescence signal intensities used to quantify clustering of proteins under the above conditions. The values represent average of at least 25 measurements. One-way ANOVA was used to demonstrate the significant differences (*p* < 0.001, “***”; *p* < 0.05, “*”; *p* > 0.05 “NS”).

Based on these results, we aimed to quantify changes in ALBA protein localization with respect to particular foci formation under standard and HS conditions. Strong protein re-localization upon HS focused our attention to Rpp25-like subfamily proteins. We selected two proteins, ALBA4-GFP and ALBA6-GFP for precise quantification of HS-induced protein clustering. The degree of protein clustering was quantified by the coefficient of variation (CV) reflecting fluctuations in fluorescence signal intensities. CV was estimated in untreated pollen and after the exposure of flowering plants to 37 °C for 3 h, both 1 h and 24 h after the treatment (Figure 7). Only optical sections with cytoplasm (without nuclei) were taken for analysis. The cytoplasmic signal for both ALBA4-GFP and ALBA6-GFP changed significantly from rather homogeneous under control conditions to a clustered/patchy distribution with clear fluorescence maxima and minima after HS (Figure 7A). Protein accumulations were more pronounced after 1 h but remained detectable 24 h after HS. Coefficient of variation of ALBA4-GFP 1 h after HS almost doubled in comparison to the untreated control (Figure 7B). After 24 h, the signal accumulation was less distinct and more dispersed in the cytoplasm. ALBA6-GFP showed a similar profile with a less apparent peak at 1 h compared to normal conditions (Figure 7B). In summation, the early stress response rapidly and statistically significantly (*p* < 0.001) enhanced both ALBA4-GFP and ALBA6-GFP protein accumulation in cytoplasmic foci, which were still visible, albeit more dispersed, 24 h after HS.

### 2.5. ALBA4 and ALBA6 Co-Localization with PABP3

ALBA family proteins are known to be associated with nucleic acids [2,3,4,5,7]. We localized all family members in the cytoplasm of pollen, and therefore searched for a suitable marker protein to determine whether they associate with mRNA. We selected one of the RNA-binding poly(A)-binding proteins (PABPs), which co-localize with mRNA in cytoplasmic granules, including stress granules [21]. In *Arabidopsis*, *PABP3,* and *PABP5* show an organ-specific expression pattern in floral organs, including the male gametophyte [22,23]. *PABP3* is natively expressed in tapetum and pollen [22]. Since the detailed native localization of PABP3 in pollen has not been studied yet, we investigated PABP3-RFP protein localization in the male gametophyte.

PABP3-RFP localizes predominantly in cytoplasmic foci equally distributed in the VC cytoplasm, enriched in the MGU region (Figure 7 and Figure S5 A1–A3). A detailed view at the MGU highlighted a reticular-like pattern around the vegetative nucleus enriched in round and elongated foci. Interestingly, the enrichment was not observed in the proximity of SC nuclei but rather tracing an area of possible SC membranes (Figure S5 B1–B3). PABP3-RFP driven by the native promoter was subjected to HS and PABP3-RFP clustering evaluated by CV analyses (Figure 7A,B). The cytoplasmic localization of PABP3 resembled ALBA4 and ALBA6′s stress-induced localization pattern.

**Figure 8:**
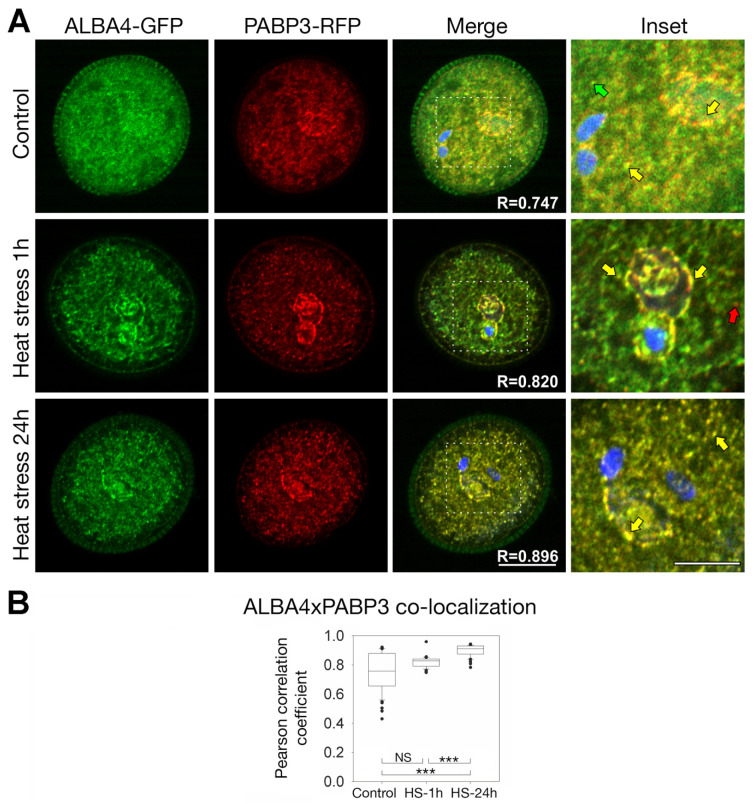
(**A**) One confocal cross-section of mature pollen expressing simultaneously ALBA4-GFP (green) and PABP3-RFP (red). DAPI staining (blue) is included in the overlay image, insets are indicated by white patchy box. The control condition (first row), 1 h (second row) and 24 h after heat stress 37 °C for 3 h (third row) are shown. The co-localization of green and red channels appears as yellow. Arrows in inset point to spots with GFP (green arrow), RFP (red arrow) and both fluorophores (yellow arrow). Scale bar 10 µm and 5 µm (inset). (**B**) Above-threshold Pearson’s correlation coefficients (R) measured from individual confocal cross sections are indicated in boxplot. The values represent average of at least 35 measurements. One way ANOVA shows statistically significant differences (*p* < 0.001, “***”; *p* > 0.05 “NS”).

Due to similarities in localization of ALBA4-GFP, ALBA6-GFP, and PABP3-RFP, we decided to analyze their co-localization. To this effect, we used plants co-expressing ALBA4-GFP or ALBA6-GFP and PABP3-RFP. Our analysis revealed a strong signal overlap between ALBA4-GFP and PABP3-RFP in the VC cytoplasm (Figure 8). The co-localization occurs in distinct foci and suggests ALBA4 association with cytoplasmic mRNA-containing particles (mRNPs) (Figure 8A) [7,21]. The degree of correlation between signal intensities was quantified by above threshold Pearson´s correlation coefficient (PCC) R (Figure 8B) measured from all optical sections in the z-stack. A significant correlation at control conditions (R = 0.747) was observed. The signal overlay becomes even more apparent after heat stress (R = 0.820) and increases in time in VC cytoplasmic foci and around MGU (Figure 8A). The maximum correlation is observed in samples collected 24 h after the stress treatment (R = 0.896). Data variability of the untreated samples originates at more or less ALBA4-GFP dispersed cytoplasmic localization. Protein clustering of ALBA4 after HS further increases the correlation between ALBA4-GFP and PABP3-RFP signals, with this trend more pronounced 24 h after HS.

**Figure 9:**
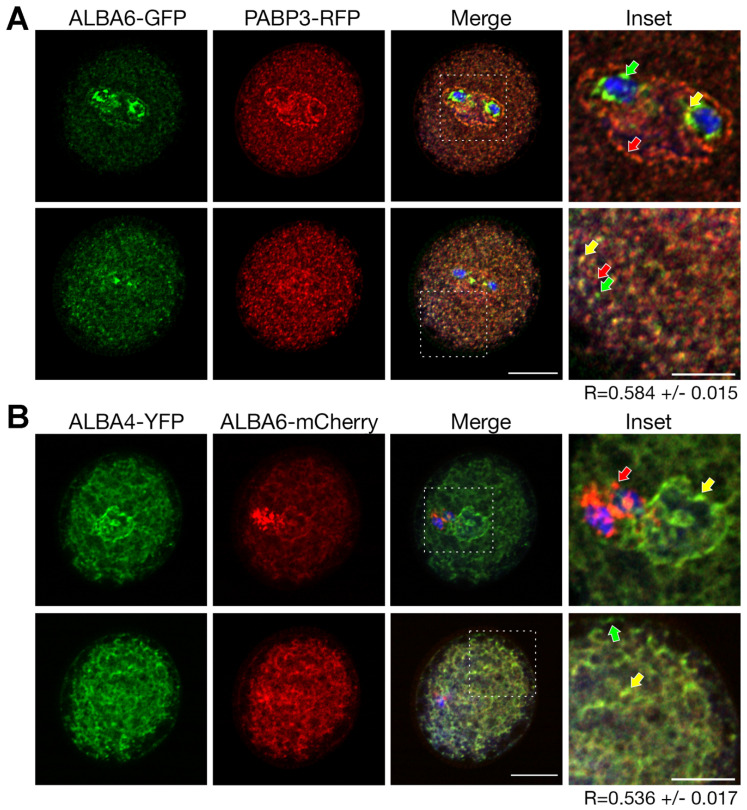
Co-localization analysis of ALBA6. (**A**) Confocal microscopy of cells expressing simultaneously ALBA6-GFP (green) and PABP3-RFP (red). Overlay of both channels includes also DAPI signal (blue). In upper row, cross-section in male germ unit (MGU) region; in lower row, cytoplasmic cross-section is presented. Insets are indicated in the overlay image by white dotted box. Arrows in inset point to spots with GFP (green arrow), RFP (red arrow) and both fluorophores (yellow arrow). Scale bar 10 µm and 5 µm (inset). Above-threshold Pearson’s correlation coefficient (R) values measured from all individual confocal cross sections are indicated. The values represent average of at least 30 measurements. (**B**) Confocal microscopy of cells expressing simultaneously ALBA4-YFP (green) and ALBA6-mCherry (red). Overlay of both channels includes also DAPI signal (blue). In upper row, cross-section in MGU region; in lower row, cytoplasmic cross-section is presented. Insets are indicated in the overlay image. Arrows in inset point to spots with GFP (green arrow), RFP (red arrow) and both fluorophores (yellow arrow). Scale bar 10 µm and 5 µm (inset). Above-threshold Pearson’s correlation coefficient (R) values measured from all individual confocal cross sections are indicated. The values represent average of at least 40 measurements.

Pollen of heterozygous ALBA6-GFP/PABP3-RFP plants was used for further co-localization analysis. The ALBA6-GFP signal is strongly enriched around SC nuclei where it rarely overlaps with the red signal of the mRNA marker (Figure 9A, upper row; inset, green and yellow arrow). The strongest red signal was detected around VN (Figure 9A, upper row; inset, red arrow) and surrounding likely SC membranes. Here it partially overlaps with ALBA6-GFP, however, with low signal intensity correlation. The weak signal overlap may occur also in the SC cytoplasm. Partial correlation of signals was identified in the VC cytoplasm (Figure 9A, lower row; inset). The average co-localization measured as PCC (including all pollen regions from the z-stack) was significantly lower (R = 0.58) than in case of ALBA4.

To complete the co-localization analysis, we also checked whether ALBA4 and ALBA6 co-localize. Transcriptional units of ALBA6-mCherry and ALBA4-YFP driven by their native promoters were combined into a single destination vector, and the signal overlap evaluated in mature pollen (Figure 9B). ALBA4-YFP was enriched in close proximity to the VC nuclear membrane, whereas ALBA6-mCherry concentrated around SC. The signal overlap was weak in the MGU region but cytoplasmic distribution of both green and red signals was similar. The partial co-localization occurred in the VC cytoplasm (Figure 9B, lower row). The overall PCC reflecting correlation of both signals in the whole cell was 0.536, which is consistent with the previous two co-localization results, suggesting different types of mRNA regulation exerted by ALBA4 and ALBA6.

## 3. Discussion

In flowering plants, the male gametophyte represents extremely reduced model for studies of molecular and cytological events leading to cell specification [17,24]. This process includes precise regulation and timing of events controlled by specific gene expression as demonstrated by transcriptome and translatome dynamics during pollen development [25,26]. There, number of active genes rapidly decreases after pollen mitosis II and pollen transcriptome becomes less complex and more specific [26]. In this study, we investigated the expression patterns and dynamics of *Arabidopsis ALBA*-family genes in developing reproductive tissues and their modulation by heat stress.

GUS assays disclosed the activity of all six *ALBA* promoters, revealing that all are active in bicellular pollen, the last stage in pollen development, with VC proliferation and differentiation activities [26]. Moreover, our observations extended and supplemented previously published data on *ALBA* expression patterns in *Arabidopsis* sporophytic tissues, with exclusive expression in developing and metabolically active tissues, root tips and young leaves [12].

During the final phases of pollen maturation, metabolic activity of vegetative cell decreases and accumulates reserves including the majority of pollen mRNA in the form of cytoplasmic ribonucleoprotein particles, or mRNP granules, of distinct protein composition in mature pollen [25,27,28,29,30,31]. Localization experiments revealed GFP fluorescence only in the mature pollen cytoplasm, particularly in regions surrounding the vegetative nucleus and sperm cells. Our observation revealed subcellular reticular-like pattern and characteristic cytoplasmic foci in pollen. These results are in keeping with previous published results on ALBA signal distribution in root cells [12]. The observed co-localization of ALBA proteins with PABP3, and the reported presence of storage particles in pollen [25,28,30], further suggests the involvement of ALBA proteins in mRNA storage in pollen, or in the translational or stability control of their target transcripts.

In rice, *ALBA* stress-responsive expression changes were detected in a wide range of tissues, under various abiotic stresses [10]. In this study, we focused on HS as an example of abiotic stresses causing the reduction of pollen viability and fitness [16]. We quantified the dynamics of *ALBA* expression in generative tissues upon HS by RT-qPCR and detected significant changes in expression levels of most *ALBA* genes in inflorescences after the application of mild HS (37 °C). On the contrary, moderate HS (42 °C) had no significant effect on *ALBA* expression. These findings suggest that *ALBA* genes are regulated in a temperature-dependent manner, and are likely involved in mild temperature stress response in *Arabidopsis*. As such, *ALBA* genes could act as thermomemory-associated genes [32] in the male gametophyte. This temperature-dependent modulation of *ALBA* expression led us to investigate ALBA-GFP localization in response to heat stress in mature pollen. The application of mild HS (37 °C for 3 h) affected ALBA-GFP localization, especially for ALBA4 and ALBA6, the two more divergent Rpp25-like subfamily members, with the most stable signal localization pattern. Similar stress-triggered re-localization of ALBA proteins between the cytoplasm and cytoplasmic foci were reported in *Trypanosoma* and *Plasmodium* [7,8].

We further traced mRNA distribution in pollen by RNA-binding protein PABP3-RFP [22,33] with no significant variation of the RFP signal after mild HS. PABPs were reported to interact with ALBA proteins in *Leishmania* [6], *Plasmodium* [7] and *Trypanosoma* [8,34] in stress granules [8]. Therefore, ALBA4 and ALBA6 co-localization with PABP3 in the VC cytoplasm is in accordance with the ALBA distribution reported in other systems. The nucleic binding capability of most *Arabidopsis* ALBAs, including ALBA4 and ALBA6 [15], their cytoplasmic co-localization with PABP3-RFP marker, and their re-localization following HS collectively support the function of these proteins in heat stress-modulated mRNA metabolism. Similar role of ALBA proteins was described in *Trypanosoma* [8], *Toxoplasma* [35], and *Leishmania* [6]. The increased co-localization of ALBA4-GFP and PABP3-RFP at elevated temperatures indicates its likely role in RNA metabolism, storage and translation regulation in pollen, in relation to heat stress response and cell homeostasis [7,19,31,36]. On the other hand, ALBA6-GFP and PABP3-RFP co-localization revealed a significantly lower signal overlap. It suggests the possible functional difference of ALBA4 and ALBA6 that is also supported by their even weaker co-localization in pollen.

Collectively we demonstrated the involvement of ALBA-family proteins in male reproductive development and in the heat stress response. Moreover, we suggest that ALBA4 and ALBA6 are implicated in RNA metabolism and storage in pollen upon heat stress. These two members (and possibly ALBA proteins in general) can dynamically re-localize between various types of mRNA-containing granules within the cytoplasmic mRNPs continuum in a controlled, developmentally and environmentally regulated manner. Such regulation then reflects not only their redundancy but also their possible functional diversification in plants. 

## 4. Materials and Methods

### 4.1. Sequence Motif, Phylogenetic, and Transcriptomic Analyses

All DNA, cDNA, and protein sequences used were obtained from TAIR (https://www.arabidopsis.org/). Motif analysis was performed using the MEME suite 5.2.0 [37]. The MEME search was set to identify a maximum of 15 motifs per protein, with maximum and minimum length of the conserved motif being 50 and 6, respectively.

Amino acid sequences were aligned using the Clustal Omega algorithm [38] in the Mobyle platform [39] with homology detection by HMM±HMM comparisons [40]. The evolutionary relationships among ALBA family members was inferred using the Maximum Likelihood method based on the JTT matrix-based model [41]. Initial trees for the heuristic search were obtained by the Maximum Parsimony method, with 1000 bootstrap replicates applied. The tree is drawn to scale, with branch lengths measured in the number of substitutions per site. The analysis involved 9 amino acid sequences. A total of 623 positions were present in the final dataset. Evolutionary analyses were conducted in MEGA7 [42]. Whole ALBA mRNAs were in silico translated and domains aligned at Cathdb.info/version/v4_2_0/domain. Schematic visualization of inflorescence ALBA proteins was designed using https://prosite.expasy.org/cgi-bin/prosite/mydomains/.

Transcriptomics data for gene expression analysis were obtained from the CoNekT online Database (www.evorepro.plant.tools) [43].

### 4.2. Cloning and Plant Transformation

For promoter fusion constructs, proALBA::GUS-GFP, native promoter sequence fragments of all ALBA genes were amplified from genomic DNA using specific primers (Table S2; Primers 17–40), and cloned into the Gateway-compatible pKGWFS7 binary vector [42,44]. For protein fusion constructs, proALBA:ALBA::GFP, full-length gene fragments of *ALBA1*-*ALBA5* containing coding regions and their native promoters were amplified from genomic DNA using specific primers (Table S2; Primers 17–40) and cloned into the Gateway-compatible pFASTR07 binary vector [44,45]. *ALBA4* (genomic DNA) and *ALBA6* (*ALBA6-4* isoform amplified from pollen cDNA) gene fragments were amplified from pollen cDNA and cloned using the GoldenBraid 3.0 system into the pDGB3 ω2 destination binary vector [46]. *ALBA4* and *ALBA6* coding regions were domesticated into pUPD2 entry vector [47] with gene specific primers (Table S2; Primers 41–56). Transcriptional units comprising native promoters and C-terminal GFP/YFP/mCherry fusions were composed in pDGB1 α11 or α12 [46]. The transcriptional units were subsequently combined in pDGB3 ω2 vector (pDGB3 ω2 ALBA6-GFP, pDGB3 ω2 ALBA6-mCherry/ALBA4-YFP) possessing a plant selection cassette.

For the co-localization of ALBA4-YFP and ALBA6-mCherry, we used GoldenBraid cloning to combine both transcriptional units into a single destination vector [46,47]. All vectors were verified by sequencing (Table S2; Primers 57–105) and used for *Agrobacterium tumefaciens*, strain GV3101 (pMP90RK) transformation. Stable transgenic plants were obtained by floral dipping [48]. Seeds of primary transformants were screened for resistance to Hygromycin B Gold™ (InvivoGen, Toulouse, France) (ALBA-GFP) or Kanamycin (Duchefa Biochemie B. V, Haarlem, The Netherlands) (proALBA::GUS-GFP and ALBA6-mCherry/ALBA4-YFP). Plants for PABP3 co-localization were derived by crossing PABP3-RFP plants with ALBA-GFP plants. All measurements were done on T1 (ALBA-GFP and PABP3-RFP) and T2 (proALBA::GUS-GFP, ALBA-GFP, PABP3-RFP, ALBA6-mCherry, and ALBA4-YFP) generations where the signal was sufficient for microscopy. More than 20 plants were screened for each obtained construct in the T1 generation to identify transgenic plants with the most similar patterns of expression and protein localization.

To prepare an *Arabidopsis* stable line expressing a tagged version of PABP3, the whole genomic sequence encompassing PABP3 (AT1G22760) (starting from position—1884 from ATG to the last nucleotide before stop codon and including introns and 5’-UTR) was PCR amplified using primers eb1 & eb2 (Table S2; Primers 17–40). The PCR fragment was subsequently restriction cloned into CTL587 (pCAMBIA-1300 derivative) upstream to the RFP tag, using KpnI and SalI, giving rise to the pEB18 plasmid. Transgenesis was performed through floral dipping of *pab3-1* (verified T-DNA insertion line SAIL_783_D04, Table S2; Primers 89–105) *Arabidopsis* flowers. Primary transformant seeds were screened for resistance to Hygromycin B Gold™.

### 4.3. Plant cultivation and Treatment

*Arabidopsis thaliana* accession Columbia-0 (Col-0) were germinated on 0.5× Murashige and Skoog (MS) medium containing 5% (w/v) Sucrose, 25% (w/v) MES and 0.8% (w/v) Agar [49], and transferred to soil after 10 days. Plants were cultivated using a long day regime at 22 °C for 3–4 weeks. Flowering plants were used for Floral dipping [48]. Transformed plants were selected on 0.5× MS medium with Hygromycin B Gold™ (Gateway vectors) or Kanamycin (GoldenBraid 3.0 vectors) for 10 days. Seedlings were transferred to soil and cultivated under standard conditions in a growth chamber (22 °C, 60% RH, 16 h light). Heat shocks were performed on flowering plants grown under standard conditions. The applied treatments were 42 °C for 1 h (70% RH, light) and 37 °C for 3 h (70% RH, light). Following stress treatments, plants were moved back to the growth chamber for 1 h and 24 h.

### 4.4. RNA Isolation and RT-qPCR

Total RNA was extracted from inflorescences collected 1 h and 24 h after heat stress application (37 °C for 3 h and 42 °C for 1 h) using the RNeasy Mini Kit (Qiagen, Valencia, CA, USA). Four biological replicates (inflorescences) were used for each experiment. RNA quantity and quality (purity) was determined using NanoDrop One (Thermo Fisher Scientific, Waltham, MA, USA). RNA quality was verified by electrophoresis in a 2% agarose gel. All samples were treated by RQ1 RNase-Free DNase (Promega, Madison, WI, USA). Reverse transcription (RT) was performed for 1 h at 42 °C using ImProm-II™ Reverse Transcription System (Promega, Madison, WI, USA) with an oligo-d(T)_20_ primer. RT-qPCR measurements were obtained using GoTaq Q-PCR Master Mix (Promega, Madison, WI, USA) on a LightCycler 480 (Roche, Basel, Switzerland) with gene specific primers (Table S2; Primers 1–16). Ct values were normalized according to *GAPC1* (At3g04120) and *EIF1a4* (At5g60390) expression levels and statistics calculated using R [50]. The data were tested for normal distribution and evaluated by Student’s t-test or the Wilcoxon test.

### 4.5. GUS Activity

The GUS staining protocol was adapted to the individual samples, with two solutions used for the final staining using the appropriate time in between 7 and 24 h. Inflorescences of T2 generation *proALBA::GFP-GUS* transgenic plants were stained for GUS activity with a solution containing 50 mM sodium phosphate buffer, pH 7, 0.2% Triton X-100, 1 mM X-Gluc (5-bromo-4-chloro-3-indolyl-D-glucoronic acid, Thermo Fisher Scientific, Waltham, MA, USA ) and 0.5 mM potassium ferrocyanide, 0.5 mM potassium ferricyanide or 2.5 mM potassium ferrocyanide, 2.5 mM potassium ferricyanide. Samples were vacuum-infiltrated for 10 min and stained at 37 °C. The staining was fixed in 99% ethanol for 24 h after which it was replaced by 70% ethanol at 4 °C.

### 4.6. DAPI Staining

Pollen from all samples was collected and stained with 0.8 µg/mL DAPI (4′-6-Diamidino-2-phenylindole, Merck KGaA, Darmstadt, Germany) in GUS buffer for 15 min (mature pollen) or 1 h (microspores, bicellular pollen) [51]. Stained samples were prepared for light and confocal microscopy.

### 4.7. Light Microscopy

Fixed *proALBA::GUS-GFP* inflorescences, stamens with unopened and opened anthers, pistils from unopened and opened flowers and young siliques were collected and captured by a Zeiss stereomicroscope (Carl Zeiss, Jena, Germany). Nectaries, ovules, seeds, microspores, bicellular and mature pollen grains were collected and captured by a widefield fluorescence microscope Zeiss Axioimager (Carl Zeiss, Jena, Germany).

Pollen grains of transgenic plants carrying ALBA-GFP and PABP3-RFP, ALBA6-mCherry and ALBA4-YFP and GUS-GFP were collected on a slide containing the DAPI staining solution and imaged under the inverted confocal laser scanning microscope Zeiss LSM880 (Carl Zeiss, Jena, Germany) equipped with an Airyscan detector and Plan-Apochromat 100×/1.46 Oil objective. For excitation of DAPI, GFP, RFP/mCherry laser lines 405 nm, 488 nm and 561 nm were used in a sequential scanning setup. Within an experiment, all images were acquired using adjusted settings reflecting various signal intensities of the individual fusion proteins. Airyscan Processing of raw files was performed in ZEN black software (Carl Zeiss, Jena, Germany).

### 4.8. Microscopy Data Analysis

Microscopy data were analyzed in the ZEN blue 2.5 software (Carl Zeiss, Jena, Germany)—Maximum intensity projection, 3D-view, and colocalization analysis. Coefficient of variation (CV) was estimated as described in [52] and modified for z-stack. Briefly, CV was calculated as the ratio of standard deviation of fluorescence intensities to the mean florescence signal, taking into account all fluorescence intensities of individual cross-sections after background subtraction. These CVs were used as measures for local fluctuations of fluorescence intensity reflecting formation of protein accumulation after heat shock.

Pearson’s correlation coefficients were calculated in Zen blue SW from each thresholded cross-section of the z-stack. Average values from both analyses were depicted as box plots, their statistical significance was calculated using One Way ANNOVA followed by Pairwise Multiple Comparison Procedures (Dunns method) in Sigma Plot (Systat, Chicago, IL, USA).

## Data Availability

Not applicable.

## Supplementary Materials

Supplementary materials can be found at https://www.mdpi.com/1422-0067/22/4/1652/s1.

## Abbreviations

ALBA	Acetylation lowers binding affinity
CV	Coefficient of variation
HS	Heat stress
GFP	Green fluorescent protein
GUS	β-glucuronidase
MGU	Male germ unit
ML	Maximum likelihood
mRNP	Messenger ribonucleoprotein particle
PABP	Poly(A)-binding protein
PABP3	Poly(A)-binding protein 3
PCC	Pearson correlation coefficient
RFP	Red fluorescent protein
Rpp20	Ribonuclease P protein subunit p20
Rpp25	Ribonuclease P protein subunit p25
SC	Sperm cell
VC	Vegetative cell
YFP	Yellow fluorescent protein
