# Supplementary material for: Characterization of ALBA Family Expression and Localization in Arabidopsis thaliana Generative Organs
Source: Int J Mol Sci. 2021 Feb 6;22(4):1652. doi: 10.3390/ijms22041652 (PMC7914821; doi:10.3390/ijms22041652)
Supplement: Supplementary file 1 [file ijms-22-01652-s001.zip › ijms-1096749 suppl/Supplement/Supplementary Material Overview.pdf]

Supplementary Figures

Figure S1: Controls used for ALBA-GFP localization. The pollen developmental stages were isolated and stained with DAPI. proALBA6::GFP-GUS construct was used for non-specific protein localization verification. GFP-GUS fusion protein was localized in cytoplasm in all three stages (A1-C3). Wild type Col-0 pollen stages were used as a negative control (D1-F3).

Figure S2: ALBA-GFP localization in mature pollen. ALBA-GFP (A1-A6) in one optical section crossing at least one of SC nuclei is presented. The ALBA-GFP expression was driven by their native promoters. Mature pollen was stained with DAPI (B). ProALBA6::GFP-GUS construct was used as cytoplasm localization marker. Scale bars = 10μm.

Figure S3: Heat stress-induced relative expression changes of *ALBA* genes in inflorescences evaluated by RT-qPCR. Flowering plants were exposed to 42 °C for 3 hours; inflorescences of treated and untreated plants were collected 1 hour (A) and 24 hours (B) after the end of the heat exposure. Relative expression values were compared with the untreated samples collected at the same time. The measurement was performed in four biological replicates and two technical replicates for each sample. All data were tested for normality and p-values were obtained for each experiment (see Table A3). The blue and red rings represent the individual replicates; sample means of each independent group are represented by black rings with indicated standard error bars.

Figure S4: Controls used for heat stress of ALBA-GFP proteins. ProALBA6::GUS-GFP plants were subjected to mild (37 °C for 3 hours (A1-B2)) or moderate (42 °C for 1 hour (C1-D2)) heat stress. Pollen samples were collected, and DNA was stained with DAPI. The confocal microscopy was done 1 hour and 24 hours after treatments. Scale bars 10 μm.

Figure S5: Subcellular localization of PABP3-RFP in mature pollen grain (A) and, in detail, in the MGU region (B). proPABP3::PABP3-RFP was expressed under the native promoter. The proPABP3:PABP3-RFP harbouring lines were selected for stable expression of the PABP3-RFP marker. VN and SN were visualised by DAPI staining. First column scale bar = 10 μm, second column scale bar = 5 μm.

Supplementary Tables

Table S1: List of ALBA proteins in pollen. Obtained cDNA sequences of all ALBAs were *in silico* translated. The obtained sequences were aligned to the known structures (UniProt; https://www.uniprot.org/) and searched for domain positions (http://www.cathdb.info/). Lengths of predicted proteins are included.

Table S2. List of primers used for Q-PCR (Primers 1-16). List of cloning primers (Primers 17-40). List of primers used for GoldenBraid cloning (Primers 41-56). List of primers used for vectors sequencing (Primers 57-88). List of primers used for cDNA amplification and mutant verification (Primers 89-105).

Table S3. Ct values of ALBA genes expression in Col-0. Inflorescences of Col-0 plants were collected 1 hour and 24 hours after heat stress (42 °C for 1 hour or 37°c for three hours) in four independent biological replicas. The RT-pPCR was provided in two technical replicas for each sample including reference genes GAPC1 and eIF1a.

Table S4. Statistical evaluation of the heat shock experiments results. We applied two stress conditions (37 °C and 42 °C) to Col-0 flowering plants. Inflorescences were collected 1 hour and 24 hours after the treatment. RT-qPCR results of individual *ALBA* genes are showed. Two technical replicates and 4 biological replicates were used for the final evaluation by Student´s t-test or Wilcoxon test and p-values of all measurements are in the right panel.

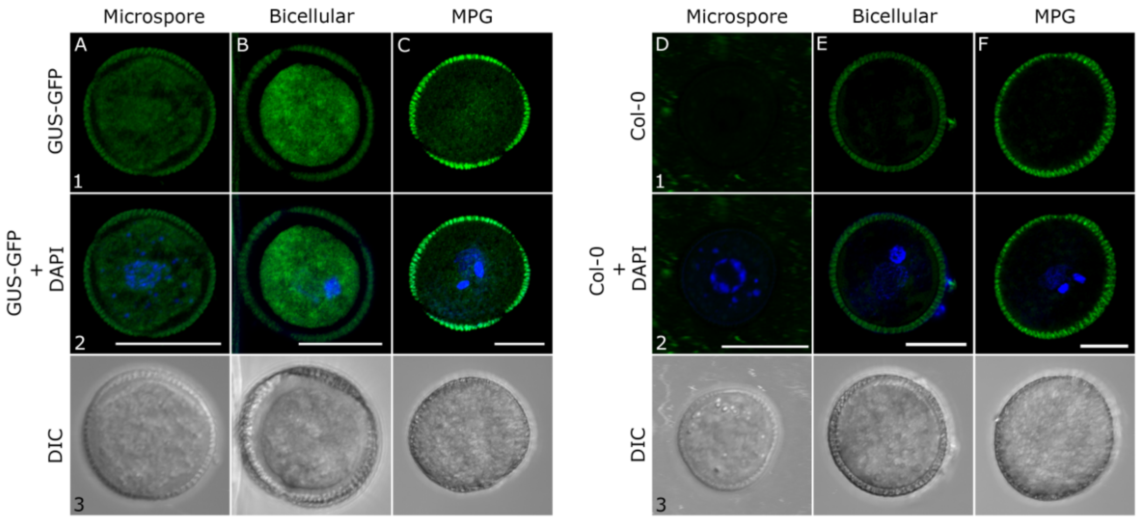

Figure S1: Controls used for ALBA-GFP localization. The pollen developmental stages were isolated and stained with DAPI. proALBA6::GFP-GUS construct was used for non-specific protein localization verification. GFP-GUS fusion protein was localized in cytoplasm in all three stages (A1-C3). Wild type Col-0 pollen stages were used as a negative control (D1-F3).

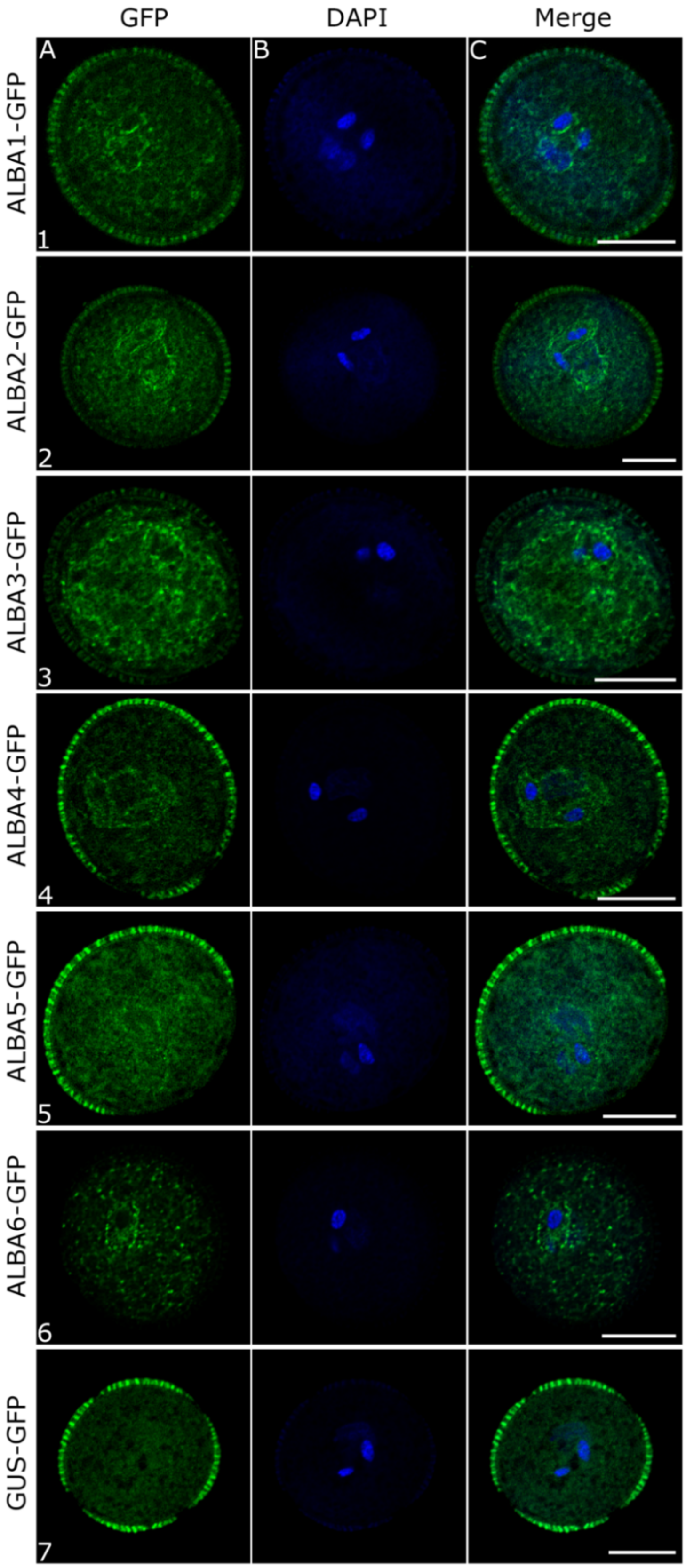

Figure S2: ALBA-GFP localization in mature pollen. ALBA-GFP (A1-A6) in one optical section crossing at least one of SC nuclei is presented. The ALBA-GFP expression was driven by their native promoters. Mature pollen was stained with DAPI (B). ProALBA6::GFP-GUS construct was used as cytoplasm localization marker. Scale bars = 10μm.

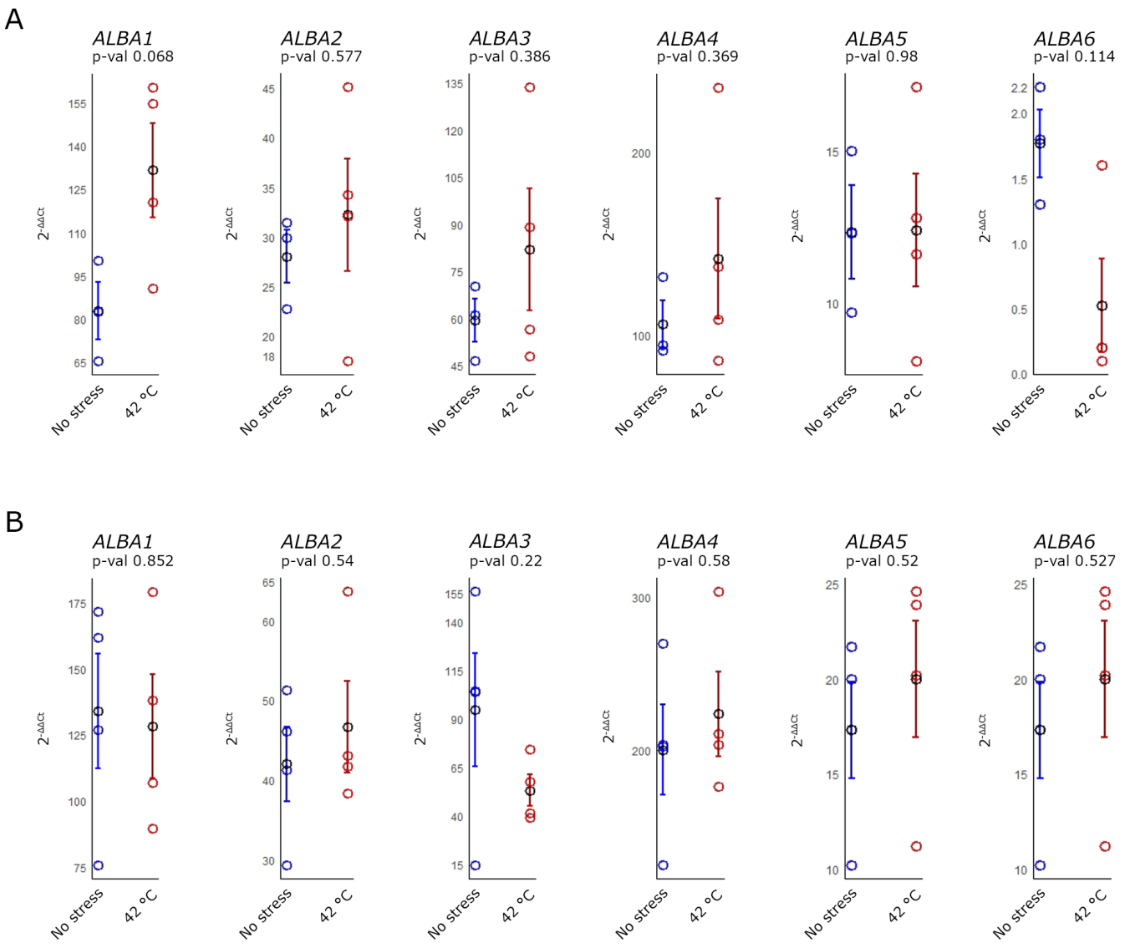

Figure S3: Heat stress-induced relative expression changes of *ALBA* genes in inflorescences evaluated by RT-qPCR. Flowering plants were exposed to 42 °C for 3 hours; inflorescences of treated and untreated plants were collected 1 hour (A) and 24 hours (B) after the end of the heat exposure. Relative expression values were compared with the untreated samples collected at the same time. The measurement was performed in four biological replicates and two technical replicates for each sample. All data were tested for normality and p-values were obtained for each experiment (see Table A3). The blue and red rings represent the individual replicates; sample means of each independent group are represented by black rings with indicated standard error bars.

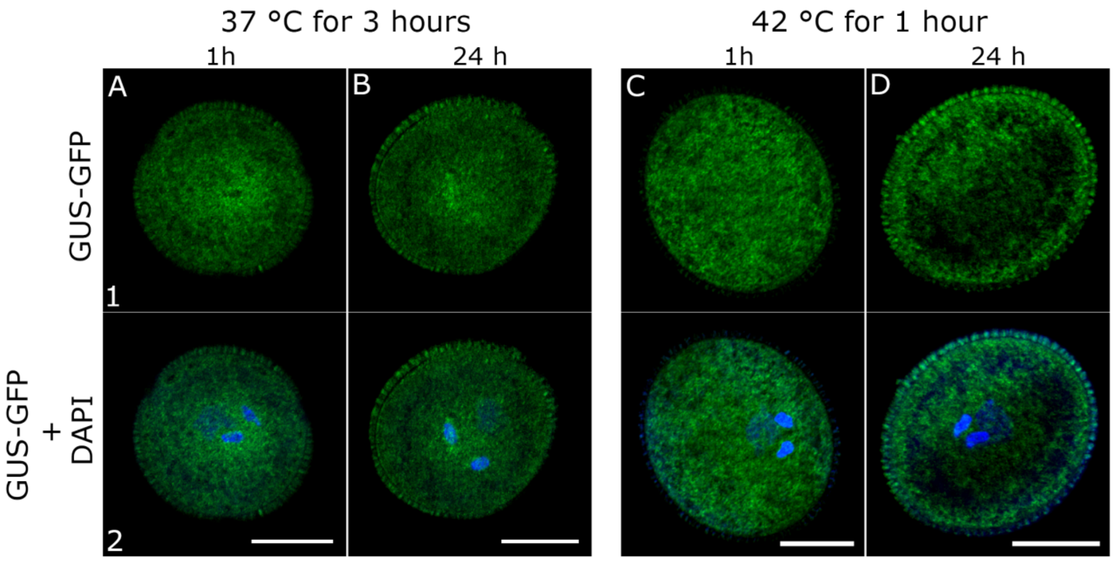

Figure S4: Controls used for heat stress of ALBA-GFP proteins. ProALBA6::GUS-GFP plants were subjected to mild (37 °C for 3 hours (A1-B2)) or moderate (42 °C for 1 hour (C1-D2)) heat stress. Pollen samples were collected, and DNA was stained with DAPI. The confocal microscopy was done 1 hour and 24 hours after treatments. Scale bars 10 μm.

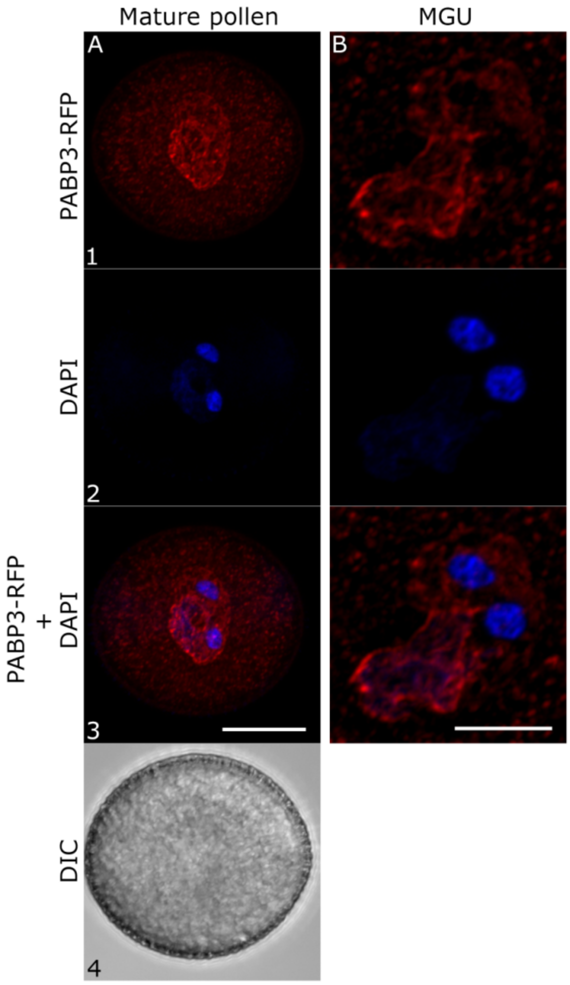

Figure S5: Subcellular localization of PABP3-RFP in mature pollen grain (A) and, in detail, in the MGU region (B). proPABP3::PABP3-RFP was expressed under the native promoter. The proPABP3:PABP3-RFP harbouring lines were selected for stable expression of the PABP3-RFP marker. VN and SN were visualised by DAPI staining. First column scale bar = 10 μm, second column scale bar = 5 μm.
